# A vertebrate case study of the quality of assemblies derived from next-generation sequences

**DOI:** 10.1186/gb-2011-12-3-r31

**Published:** 2011-03-31

**Authors:** Liang Ye, LaDeana W Hillier, Patrick Minx, Nay Thane, Devin P Locke, John C Martin, Lei Chen, Makedonka Mitreva, Jason R Miller, Kevin V Haub, David J Dooling, Elaine R Mardis, Richard K Wilson, George M Weinstock, Wesley C Warren

**Affiliations:** 1 The Genome Center, Washington University School of Medicine, Campus Box 8501, 4444 Forest Park Avenue, St Louis, MO 63108, USA; 2 The J Craig Venter Institute, 9712 Medical Center Drive, Rockville, MD 20850, USA

## Abstract

The unparalleled efficiency of next-generation sequencing (NGS) has prompted widespread adoption, but significant problems remain in the use of NGS data for whole genome assembly. We explore the advantages and disadvantages of chicken genome assemblies generated using a variety of sequencing and assembly methodologies. NGS assemblies are equivalent in some ways to a Sanger-based assembly yet deficient in others. Nonetheless, these assemblies are sufficient for the identification of the majority of genes and can reveal novel sequences when compared to existing assembly references.

## Background

Whole genome assemblies are defined as hierarchical structures of sequence units, or 'contigs', built from overlapping sequence reads, that are linked together physically into higher order 'supercontigs'. How completely one can reconstruct the genome of a species *de novo *is dependent on a number of genomic properties, including repeat content, heterozygosity and ploidy, as well as the sequencing platform used to generate the primary data. Over the past decade most large (>1 Gbp) genomes were sequenced exclusively on capillary-based Sanger sequencers. The emergence of next-generation sequencing (NGS) technologies has led to the promise of rapidly generating *de novo *genome assemblies for a wide variety of species, including vertebrates with large complex genomes. Although the use of NGS data is now an established paradigm for producing microbe assemblies, constructing highly contiguous assemblies using NGS data from higher organisms has been challenging [1,2]. Li *et al*. [1] generated independent *de novo *assemblies of two human genomes, and the more contiguous of these two covered 95% of the human reference. In the latest example, Gnerre *et al*. [3] generated even higher contiguity human and mouse assemblies using a spectrum of library types sequenced on the Illumina platform. Despite these advances, many questions remain about the optimal NGS data mixture required to reach contiguity goals, making chromosomal assignments from NGS contigs and supercontigs, and the effect of NGS assemblies on gene annotation, among others.

Sequencing technology development continues its rapid pace with great promise for significant cost savings for *de novo *projects [4]. The most prevalent commercially available NGS instruments include the Roche 454 Life Sciences Genome Sequencer FLX [5], Applied Biosystems SOLiD [6], and the Illumina Inc. Genome Analyzer (GA) *IIx *and HiSeq 2000 [7]. Read lengths for NGS platforms range from 50 to 400+ bp, with data volumes measured in the hundreds of megabases to well over a gigabase per run, in both fragment and paired end configurations. In fact, the sheer volume of NGS data is a significant challenge to assembly algorithm development in areas where computer memory may be limited. Library insert size is also variable, with several long-span paired-end library protocols available [3].

To assemble short read types, numerous algorithms have been developed that rely on graph-based progression [8]. Some of these algorithms were specifically developed to avoid the problems faced when trying to apply traditional overlap-layout-consensus methods to NGS data: short and numerous read overlaps, particularly for Illumina/SOLiD data, that prove to be too computationally demanding [9,10]. Most all of these algorithms rely on the de Bruijn graph method [11]. For longer 454 reads, the de Bruijn graph method and the traditional overlap-layout-consensus approach are used with specific modifications, such as filtering partial adaptor sequences, homopolymer runs, and redundant read pairs [12,13]. Regardless of which sequencing technology is used, how well the assembly algorithm addresses the inherent weaknesses of each technology determines, in large part, the assembly quality.

With the relatively simplistic structure of the chicken genome [14] and estimated size of 1.2 Gbp, we hypothesized it would serve as an optimal assembly model. Pertinent advantages include the many available validation resources, such as the current reference assembly, 193 finished BACs, and gene annotations. Importantly, each of these resources was generated from the same DNA source used to generate the NGS data, allowing for a true apples-to-apples assessment of assembly quality.

## Results

### Sequencing

**Figure 1:**
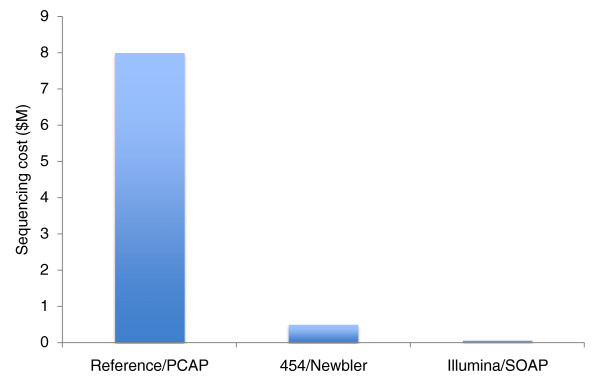
**Sequencing cost of NGS assemblies compared to the reference assembly**. The coverage of raw bases for the reference assembly is 6.6-fold, for the 454/Newbler assembly 14-fold, and for the Illumina/SOAP assembly 74-fold.

The DNA source was the original female red jungle fowl bird (UDC 001). A total of 14-fold sequence coverage of 454 (FLX and Titanium) and 74-fold coverage of Illumina (GA *IIx*) data was generated (Table S1 in Additional file 1). The targets for total 454 read coverage were modeled after our experiments with the *Caenorhabditis elegans *genome, whereas the Illumina coverage model followed the recommendations of an earlier report [1,2]. Read lengths varied depending on the instrument and the single versus paired-end approach to library construction. On the 454 platform, 11-fold coverage of Titanium fragment reads, 1-fold coverage of FLX paired-end reads with 3-kbp inserts, and 1-fold coverage of Titanium paired-end reads with 20-kbp inserts were generated. On the Illumina platform, 30-fold coverage of 2 × 100-bp paired-end reads with 200-bp inserts, 32-fold coverage of 2 × 100-bp paired-end reads with 300-bp inserts, and 12-fold coverage of paired-end reads with 2-kbp inserts were generated. The cost advantage of NGS data represents a savings of 16- to 160-fold compared to legacy technology (Figure 1).

### Assembly

Assemblies were generated using Newbler (version 2.0.1) [12] and SOAPdenovo (release 1.04) [1]. The chicken reference assembly used for comparisons was produced with PCAP [14]. The total assembled bases contained in contigs greater than 100 bp was 0.98 Gbp and 1.00 Gbp for the 454/Newbler and Illumina/SOAP assemblies, respectively.

### Contiguity

Contiguity statistics were computed for all assemblies (Table 1). The reference assembly demonstrated the highest contiguity statistics with a N50 contig length of 45 kbp and N50 supercontig length of 11 Mbp. The N50 statistic is defined as the largest length, *L*, such that 50% of all nucleotides are contained in contigs/supercontigs of size at least *L*. With a negligible difference in assembled genome size (1.0 Gbp 454/Newbler versus 0.98 Gbp Illumina/SOAP), contig N50 lengths for the 454/Newbler and Illumina/SOAP assemblies were 2.8- and 3.7-fold lower than the reference, respectively (Table 1). Supercontig N50 lengths for the 454/Newbler and Illumina/SOAP assemblies were 18.9- and 35-fold lower than the reference, respectively (Table 1). In general, we observed greater fragmentation and smaller contig and supercontig N50s in NGS assemblies.

**Table 1:** Comparative assembly contiguity and accuracy measures

Metric	Reference	454/Newbler	Illumina/SOAP
Q20 coverage (×)	6.1	11.1	68.6
N50 contig (kbp)	45	16	12
N50 supercontig (kbp)	11,000	584	314
BAC coverage (%)	98.4	96.0	95.6
Gene coverage (%)	97.7	93.0	93.5
Substitution rate (%)	0.0174	0.0179	0.0073
Deletion rate (%)	0.0009	0.034	0.0005
Insertion rate (%)	0.0012	0.0049	0.0002

### Accuracy

We estimated accuracy by aligning each NGS assembly to finished BACs and examining single base substitution, insertion, and deletion rates (see Materials and methods). We observed that the single base substitution rates were low (<0.02%) for all assemblies (Table 1). Moreover, the insertion rates were even lower (< 0.005%) regardless of assembly type. In contrast, the 454/Newbler assembly showed a considerably higher deletion rate of 0.034% (Table 1).

We also estimated the rate of mis-assembled contigs from the NGS assemblies relative to the reference assembly by identifying contigs that were uniquely aligned to more than one chromosome, within sequence length cutoffs. Alignments shorter than the defined cutoffs were not considered. When normalized to average supercontig length, the Illumina/SOAP assembly demonstrated fewer mis-assembly events compared to the 454/Newbler assembly at all measured size cutoffs (10 to 50 kbp; Table 2). While most are true mis-assemblies, as seen when they are examined manually, some could be examples of contigs mis-ordered in the reference assembly. However, the BAC fingerprint map, genetic linkage and radiation hybrid maps, mRNA information, and extensive manual annotation have been incorporated into the reference assembly [14], making incorrect sequence placement in the reference less likely. An earlier extrapolation of predicted mis-ordered contigs within the chicken reference was less than 0.1% [14]. Additionally, we mapped an independent set of sequence data from a 2-kbp paired-end Illumina libary to each assembly using BWA [15]. Of 126.6 million pairs total, 48.7 million aligned properly to the reference assembly, 44.6 million to the 454/Newbler assembly, and 47.5 million to the Illumina/SOAP assembly. The results showed that NGS assemblies generally have more unmapped pairs, but a higher level of fidelity in the Illumina/SOAP assembly. As expected, the majority of mis-assembly events in the 454/Newbler and Illumina/SOAP assemblies that were visually inspected were due to repeat structure flanking unique sequence.

**Table 2:** Mis-assembly events for various length cutoffs normalized to average supercontig length

Mis-assembly size (kbp)	454/Newbler	Illumina/SOAP
10	31 (51)	6 (7)
25	8 (25)	3 (6)
50	6 (22)	1 (3)

Values in parentheses are mis-assembly events before normalization.

### Genome representation

The percentage of test assembly bases aligned to finished BACs, considered to be the highest quality reference due to the use of robust base calling error models, manual local assembly inspection and a haploid DNA source, was evaluated as a measure of genome representation. Using a set of 193 finished autosomal BAC sequences (38 Mbp), derived from the same DNA source as the reference, the reference assembly covered 98.4% of total bases, while the NGS assemblies covered 96.0% (454/Newbler) and 95.6% (Illumina/SOAP) of the finished BACs, respectively (Table 1).

**Figure 2:**
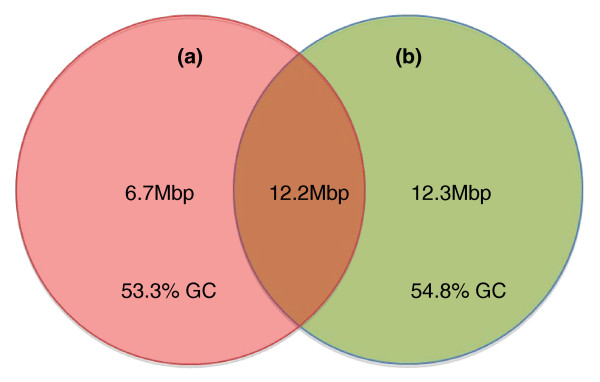
**Novel sequence in NGS assemblies compared to the reference assembly**. Each assembly was aligned to the Gallus_gallus-2.1 reference using BLAT and unaligned sequence was retained. After contamination removal, the 454/Newbler and Illumina/SOAP assemblies contain 18.9 Mbp and 24.5 Mbp of novel sequence, respectively. The NGS assemblies shared 12.2 Mbp of the non-reference sequence. **(a) **454/Newbler (red); **(b) **Illumina/SOAP (green).

The NGS assemblies were then aligned to the reference assembly using BLAT [16] with a 95% identity cutoff to evaluate whole-genome coverage and identify potential missing sequences in the reference. In this analysis multiple matches of each query were allowed. Both NGS assemblies covered 94.0% of the reference assembly. All contigs from each test assembly with no alignment to the reference were assessed for sequence content that was not captured by Sanger sequencing. The Illumina/SOAP assembly generated a total of 24 Mbp of novel sequence that have no alignment to the current reference sequence at 90% identity and 21 Mbp of non-reference sequence was found in the 454/Newbler assembly. We aligned these novel sequences against the nt database [17] with 98% identity and 200 bp length cutoff. Most aligned to recently finished chicken BAC or cDNA/mRNA sequences. In total, 81.0% of the novel sequences from the Illumina/SOAP assembly and 48.1% from the 454/Newbler assembly matched recently sequenced BACs. We found 16.3% of the novel sequences from the Illumina/SOAP assembly and 9.0% from the 454/Newbler assembly matched cDNA/mRNA sequences. Approximately 41.6% of the novel sequences from the 454/Newbler assembly that did not align to the reference were composed of contamination, as opposed to 0.4% from the Illumina/SOAP assembly. Most of the contamination (83.3%) in the 454/Newbler assembly is from *Escherichia coli*. After removing contamination, the test assemblies presented a total of 31 Mbp of putatively valid non-reference sequence, 12 Mbp of which was shared between the test assemblies (Figure 2). The average GC content of this shared non-reference portion was 54.2%, higher than the estimated 41.6% GC content for the reference genome-wide.

### Gene representation

A comprehensive evaluation of gene coverage utilized two independent gene transcript sources: 17,934 unspliced *Gallus gallus *gene transcripts from Ensembl 59 [18] and 19,626 finished cDNAs [19]. Approximately 97.7% of the total bases from the unspliced gene transcript set were present in the reference (Table 1). Both NGS assemblies cover about 93% of gene bases, which are, on average, 4% less than those covered by the reference assembly (Table 1).

**Figure 3:**
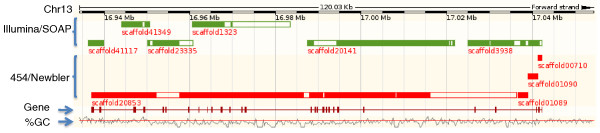
**Gene fragmentation in NGS assemblies**. Gene ENSGALG00000006569 locates from 16,937,180 to 17,042,224 on chromosome 13 in the Gallus-gallus-2.1 reference. The gene is broken into six scaffolds in the Illumina/SOAP assembly, and four scaffolds in the 454/Newbler assembly. Green bars represent scaffolds in the Illumina/SOAP assembly, and red bars represent scaffolds in the 454/Newbler assembly. Solid colored bars within scaffolds represent aligned regions while open bars denote gaps. The percentage GC (%GC) plot shows the relative GC content along the genome sequence. The horizontal red line indicates 50% GC content.

Towards an assessment of gene completeness, we evaluated the relative coverage of the reference and test assemblies across the 19k cDNA set, initially using a threshold of 90% transcript length and >95% identity to indicate successful coverage. Using these criteria, the reference contains 11% more complete cDNAs than the best NGS assembly (Additional file 2). We noted that by drastically lowering the length threshold to 20% of the cDNA length, cDNA coverage of both NGS assemblies increases approximately 20% (Additional file 2). Interestingly, at the 20% length cutoff, both NGS assemblies outperformed the reference, reflecting the fragmented nature of NGS assemblies. Most likely, the NGS assemblies are able to reveal more partial genes. As an example of the gene fragmentation observed in NGS assemblies, we mapped NGS assembly supercontigs to the reference assembly for the Rap guanine nucleotide exchange factor gene (5,083 transcript base length), located on chromosome 13. The gene is broken into six supercontigs in the Illumina/SOAP assembly, and four in the 454/Newbler assembly (Figure 3), demonstrating reduced representation in each NGS assembly.

## Discussion

Using several assembly quality metrics, the critical question we wished to address was how do *de novo *NGS assemblies compare to the Gallus_gallus-2.1 reference [14], an assembly based on well-established Sanger data. Our results have validated previous reports [1] that the assembly of large (>1 Gbp) vertebrate genomes is possible using both 454 and Illumina data. The NGS assemblies discussed herein represent advancements in our ability to assemble and analyze large genomes using NGS, further diminishing the need for solely relying on Sanger sequencing in *de novo *genome projects and presenting an opportunity to explore hybrid assemblies that utilize reads from multiple sequencing platforms, especially for existing low coverage Sanger projects.

In spite of ongoing debate on what should be the genome assembly standard in the era of NGS [20], it is encouraging that our assemblies and others derived from NGS are progressing to higher levels of contiguity and quality, and show promise in identifying novel sequences. This study is the first report to measure changes in single base substitution, insertion, and deletion rates as well as contig order and orientation among NGS assemblies derived from the same DNA source as a published reference. The advantage of this approach is that we can be confident mis-assembly calls are not due to structural variation between individuals. Using discordant paired end mapping and contig alignment methods, we conclude the reference is of higher quality than either NGS assembly. Overall, our estimates of the rate of mis-assembly events within NGS assemblies, as compared to the reference assembly, show an advantage to the lower cost Illumina/SOAP assembly (Table 2). In practice, repeat element expansion and organization in the genomes of other more complex species will determine if comparable assembly accuracy is achievable. Importantly, even Sanger based draft assemblies are not complete in the accurate representation of segmental duplications but this is much more a problem in NGS assemblies [21].

It is generally accepted that the 454 sequencing method has a diminished ability to accurately measure homopolymer base stretches compared to other platforms. This manifested in our analysis as a higher deletion rate in the 454/Newbler assembly than the Illumina/SOAP assembly and the reference assembled with PCAP, despite the optimization of Newbler to handle this error model by considering flowgrams. That said, Newbler showed a lower deletion rate than the PCAP assembler when applied to the same 454 data set, most likely because PCAP does not consider flowgrams (Table S3 in Additional file 1) [9]. Interestingly, assembling a combination of Sanger and 454 reads effectively lowers the deletion rate using CABOG [13], which was optimized for assembling hybrid data (Table S3 in Additional file 1). Other post-assembly manipulation methods can also be utilized to correct deletion or insertion errors, regardless of read types [22].

The discovery of novel sequence not found in the current chicken reference assembly was another important goal of these NGS assembly experiments. The chicken genome is rich with high GC microchromosomes that are typically underrepresented by whole-genome Sanger sequencing approaches compared to the macrochromosomes [14]. These high GC regions are also known to be gene rich; thus, their under-representation is a possible culprit for initial low gene number estimates [14]. An important question, then, is whether NGS can be used to recover these GC-rich regions and other sequences not captured in existing Sanger-based draft assemblies. In this study, NGS assemblies uncovered a total of 31 Mbp of non-reference sequence with a high average GC content (54.2%) compared to the autosomal average (41.6%). It appears NGS can be a useful means to capture missing sequences in draft assemblies that were built using Sanger data. The 454 platform has also been shown to be effective in the recovery of sequences from microbial genomes with high GC content (>60% GC) [23] and in closing gaps in the human genome [24]. Furthermore, a protist genome project (*Leishmania donovani*) utilized Illumina data to close 46% of the gaps in a 454-based assembly, showing that hybrid approaches can effectively leverage the strengths of each platform [25].

In terms of gene representation, we observed approximately 93% coverage of the Ensembl gene set in both NGS assemblies, similar to the 89% of RefSeq genes covered by an all-Illumina assembly of the human genome [1]. This number does not express, however, whether gene footprints are represented contiguously, and we found evidence of high gene fragmentation in NGS assemblies when we reduced our alignment length thresholds. In support of these findings, only 70% of known human genes were found to be in one scaffold of a human sample assembled from all Illumina reads, suggesting extensive disruptions in gene contiguity [21]. Clearly, there is an increasing need for robust gene modeling algorithms that can take such fragmentation into account. Additionally, the difficulty of chromosomal assignment, ordering and orientating NGS contigs and supercontigs increases in parallel with fragmentation.

While the low repetitive content (approximately 10%) of the chicken genome [14] limits the direct modeling of assembly quality expectations for genomes with higher repeat complexity, such as mammals, there are several analyses that can be performed equally well on NGS and Sanger-based assemblies. Non-coding RNA transcripts having lengths shorter than typical NGS read and contig lengths can be readily annotated from known non-coding RNA. However, there are an equal number of limitations encountered when using these NGS assemblies. A summary of the assembly algorithm barriers and outcome confines has been presented elsewhere [21,22,26]. One example is the inability to detect the distribution of segmental duplications within the genome, considered crucibles of gene birth [21].

The intermediate-sized chicken genome (1.2 Gbp) serves as a good starting point to test and optimize algorithms prior to assembling mammalian genomes. For microbial genomes, short insert libraries are sufficient to produce high quality assemblies. When considering larger genomes, longer reads and libraries with larger insert sizes are necessary to span longer repeats. As this paper was under review, Gnerre *et al*. [3] successfully assembled mammalian genomes with greatly improved coverage and accuracy using ALLPATHS-LG. These assemblies cover about 40% of segmental duplication content, compared to about 12% in SOAP assemblies. Although the ALLPATHs-LG algorithm requires specialized libraries to assemble mammalian genomes, including long fragment, short jump, long jump and fosmid jump libraries at high coverage, and a minimum of 90-fold coverage, we are eager to test its effectiveness on a range of complex genomes.

The cost advantage of NGS [4] has already pushed whole genome sequencing budgets into a more acceptable range for numerous funding agencies, prompting an international consortium of scientists to propose sequencing 10,000 vertebrate species [27]. With the promise of even longer read lengths from evolving sequencing technology, our ability to create nearly complete genome sequences, even navigating repeat structures that have been resistant to all types of assembly methodology, is moving forward. Efforts to optimize this approach are underway in our lab and many others with the goal of increasing the utility of *de novo *assemblies in comparative and experimental studies.

## Conclusions

Here we present evidence that NGS assembly quality is sufficient to obtain coverage of the majority of genic content from a moderately sized vertebrate genome, with suitable contiguity for many genomic analyses, and uncover previously un-represented sequences. Exceptions included high deletion rates within 454-only Newbler assemblies and high gene fragmentation among all NGS assemblies compared to a Sanger-based reference. For this reason we predict the advancement and integration of long-span paired-end libraries will ultimately be needed to produce robust and highly contiguous NGS assemblies with greater coverage of entire gene footprints. Thus, users of NGS assemblies should be aware of these current benefits and limitations.

## Materials and methods

### Resources

DNA from a single female red jungle fowl (UCD 001) was used for all library construction and sequencing [14].

### Sequencing

Libraries for 454 Titanium fragment, FLX 3 kbp and Titanium 20 kbp paired-end sequencing were prepared using standard protocols (Roche 454 Life Sciences). 454 sequence reads were generated according to established methods [12]. Q20 base coverage for each read type is summarized in Table S1 in Additional file 1. Illumina sequencing was completed on the Illumina GA *IIx *instrument using standard protocols. All the reads have been deposited in the NCBI Sequence Read Archive [SRA:SRP005856].

### Assembly

The pre-released version 2.0.1 of Newbler was used to generate the 454 assembly (Roche 454 Life Sciences). The parameters for the Newbler assembly were: -large -consed and -cpu 8. SOAPdenovo version 1.04 [1] was used for the assembly of Illumina reads. The parameters for the SOAP assembly were: -K 31 -R -p 8. For the two small insert libraries, pair_num_cutoff = 4 and map_len = 35; for the large insert library, pair_num_cutoff = 5 and map_len = 35. The assemblies were performed on a computer having eight 2.9 GHz Quad-Core AMD Opteron Model 8389 processors (32 processor cores total) and 512 GB of RAM running GNU/Linux (Ubuntu 8.04 LTS).

### Quality assessment

A total of 193 finished BAC clones covering 38 Mbp were used for quality assessment of the assemblies. We compared each assembly to the finished clones to evaluate a number of metrics, including discrepancies (substitutions, deletions, insertions) and coverage. There are three major phases to the process: WU-BLASTN [28] alignment of each finished clone against the assembly contigs, refining those alignments with Cross-Match [29] using default parameters, and then calculating alignment statistics. For global mis-assembly events measured in Table 2 we aligned the NGS assemblies to the chicken reference Gallus_gallus-2.1 with BLAT [16]. Contigs that were uniquely aligned to more than one chromosome or region beyond specified sequence length cutoffs were counted as mis-assembled contigs. The total number of mis-assembled contigs at each threshold was normalized to average supercontig length. Normalization is done by:

where *N *is the number of mis-assembly events and *L *is the average supercontig length.

### Estimating amount of novel sequence

All contigs from each respective assembly were broken into 1-kbp non-overlapping segments (except the last segment of the contig; if its length was less than 1 kbp, it was searched instead as a piece of the penultimate 1-kbp segment). Each segment was aligned to the Gallus_gallus-2.1 reference using BLAT [16]. All unmapped sequences over 50 bp were considered putative novel sequences.

### Coverage of gene transcripts

Assembled contigs were fragmented into sequential 1-kbp chunks and aligned by WU-BLAST (parameters: M = 1 N = -3 R = 3 Q = 3 wordmask = seg lcmask topcomboN = 1 hspsepsmax = 100 golmax = 0 B = 250 V = 250) to the full set of 17,934 unspliced *G. gallus *gene transcripts downloaded from Ensembl 59 via BioMart [18]. This allowed us to consider only the single, best alignment per query when calculating coverage. We filtered out all alignments that did not meet a cutoff of greater than 95% identity over at least 100 bp. We then calculated the total number of bases uniquely covered across all chicken genes. Secondly, we searched 19,626 finished cDNA sequences [19] against all assemblies using BLAT (default settings) with a minimum identity of 90% at varying alignment length cutoffs.

## Abbreviations

BAC: bacterial artificial chromosome; bp: base pair; GA: Genome Analyzer; Gbp: giga base pair; kbp: kilo base pair; Mbp: mega base pair; NGS: next-generation sequencing.

## Authors' contributions

WCW conceived of the study and participated in its design and coordination. KVH developed the paired-end libraries. PM led the assembly management. ERM, GMW and RKW led the sequencing management. LY, LWH, PM, LC, NT, DPL, JCM, MM, DJD and JRM performed the data analysis. LY and WCW drafted the manuscript. All authors contributed to and approved the final manuscript.

## Supplementary Material

Additional file 1 **Tables S1 to S3 - sequence coverage and additional assembly results**. Click here for file

Additional file 2 **Figure S1 - coverage of finished cDNAs**. Coverage of finished cDNAs (19,626 sequences) as measured using BLAT. A cDNA sequence was considered as sufficiently covered if the percentage identity (95%) and length of the mapped portion was over the indicated cutoff. The alignment length cutoffs shown are 90%, 50%, and 20%. Blue represents the reference, red the 454/Newbler assembly, and green the Illumina/SOAP assembly. Click here for file

## References

[B1] Li R Zhu H Ruan J Qian W Fang X Shi Z Li Y Li S Shan G Kristiansen K Li S Yang H Wang J Wang J *De novo *assembly of human genomes with massively parallel short read sequencing. Genome Res 2009 20 265 272 10.1101/gr.097261.109 20019144 PMC2813482

[B2] Li R Fan W Tian G Zhu H He L Cai J Huang Q Cai Q Li B Bai Y Zhang Z Zhang Y Wang W Li J Wei F Li H Jian M Li J Zhang Z Nielsen R Li D Gu W Yang Z Xuan Z Ryder OA Leung FC Zhou Y Cao J Sun X Fu Y The sequence and de novo assembly of the giant panda genome. Nature 2010 463 311 317 10.1038/nature08696 20010809 PMC3951497

[B3] Gnerre S Maccallum I Przybylski D Ribeiro FJ Burton JN Walker BJ Sharpe T Hall G Shea TP Sykes S Berlin AM Aird D Costello M Daza R Williams L Nicol R Gnirke A Nusbaum C Lander ES Jaffe DB High-quality draft assemblies of mammalian genomes from massively parallel sequence data. Proc Natl Acad Sci USA 2011 108 1513 1518 10.1073/pnas.1017351108 21187386 PMC3029755

[B4] Mardis ER Next-generation DNA sequencing methods. Annu Rev Genomics Hum Genet 2008 9 387 402 10.1146/annurev.genom.9.081307.164359 18576944

[B5] 454. http://www.454.com

[B6] SOLiD. http://www.appliedbiosystems.com

[B7] Illumina. http://www.illumina.com

[B8] Miller J Koren S Sutton G Assembly algorithms for next-generation sequencing data. Genomics 2010 95 315 327 10.1016/j.ygeno.2010.03.001 20211242 PMC2874646

[B9] Huang X Wang J Aluru S Yang SP Hillier L PCAP: a whole-genome assembly program. Genome Res 2003 13 2164 2170 10.1101/gr.1390403 12952883 PMC403719

[B10] Jaffe DB Butler J Gnerre S Mauceli E Lindblad-Toh K Mesirov JP Zody MC Lander ES Whole-genome sequence assembly for mammalian genomes: Arachne 2. Genome Res 2003 13 91 96 10.1101/gr.828403 12529310 PMC430950

[B11] Pevzner PA Tang H Waterman MS An Eulerian path approach to DNA fragment assembly. Proc Natl Acad Sci USA 2001 98 9748 9753 10.1073/pnas.171285098 11504945 PMC55524

[B12] Margulies M Egholm M Altman WE Attiya S Bader JS Bemben LA Berka J Braverman MS Chen YJ Chen Z Dewell SB Du L Fierro JM Gomes XV Godwin BC He W Helgesen S Ho CH Irzyk GP Jando SC Alenquer ML Jarvie TP Jirage KB Kim JB Knight JR Lanza JR Leamon JH Lefkowitz SM Lei M Li J Genome sequencing in microfabricated high-density picolitre reactors. Nature 2005 437 376 380 16056220 10.1038/nature03959 PMC1464427

[B13] Miller JR Delcher AL Koren S Venter E Walenz BP Brownley A Johnson J Li K Mobarry C Sutton G Aggressive assembly of pyrosequencing reads with mates. Bioinformatics 2008 24 2818 2824 10.1093/bioinformatics/btn548 18952627 PMC2639302

[B14] International Chicken Genome Sequencing Consortium Sequence and comparative analysis of the chicken genome provide unique perspectives on vertebrate evolution. Nature 2004 432 695 716 10.1038/nature03154 15592404

[B15] Li H Durbin R Fast and accurate short read alignment with Burrows-Wheeler transform. Bioinformatics 2009 25 1754 1760 10.1093/bioinformatics/btp324 19451168 PMC2705234

[B16] Kent WJ BLAT - the BLAST-like alignment tool. Genome Res 2002 12 656 664 11932250 10.1101/gr.229202 PMC187518

[B17] nt. ftp://ftp.ncbi.nih.gov/blast/db

[B18] BioMart. http://www.biomart.org

[B19] Chicken cDNA. ftp://www.chick.manchester.ac.uk/pub/chickest

[B20] Chain PS Grafham DV Fulton RS Fitzgerald MG Hostetler J Muzny D Ali J Birren B Bruce DC Buhay C Cole JR Ding Y Dugan S Field D Garrity GM Gibbs R Graves T Han CS Harrison SH Highlander S Hugenholtz P Khouri HM Kodira CD Kolker E Kyrpides NC Lang D Lapidus A Malfatti SA Markowitz V Metha T Genomics. Genome project standards in a new era of sequencing. Science 2009 326 236 237 10.1126/science.1180614 19815760 PMC3854948

[B21] Alkan C Sajjadian S Eichler EE Limitations of next-generation genome sequence assembly. Nat Methods 2010 8 61 65 10.1038/nmeth.1527 21102452 PMC3115693

[B22] Meader S Hillier LW Locke D Ponting CP Lunter G Genome assembly quality: assessment and improvement using the neutral indel model. Genome Res 2010 20 675 684 10.1101/gr.096966.109 20305016 PMC2860169

[B23] Goldberg SM Johnson J Busam D Feldblyum T Ferriera S Friedman R Halpern A Khouri H Kravitz SA Lauro FM Li K Rogers YH Strausberg R Sutton G Tallon L Thomas T Venter E Frazier M Venter JC A Sanger/pyrosequencing hybrid approach for the generation of high-quality draft assemblies of marine microbial genomes. Proc Natl Acad Sci USA 2006 103 11240 11245 10.1073/pnas.0604351103 16840556 PMC1544072

[B24] Garber M Zody MC Arachchi HM Berlin A Gnerre S Green LM Lennon N Nusbaum C Closing gaps in the human genome using sequencing by synthesis. Genome Biol 2009 10 R60 10.1186/gb-2009-10-6-r60 19490611 PMC2718494

[B25] Tsai IJ Otto TD Berriman M Improving draft assemblies by iterative mapping and assembly of short reads to eliminate gaps. Genome Biol 2010 11 R41 10.1186/gb-2010-11-4-r41 20388197 PMC2884544

[B26] Schatz MC Delcher AL Salzberg SL Assembly of large genomes using second-generation sequencing. Genome Res 2010 20 1165 1173 10.1101/gr.101360.109 20508146 PMC2928494

[B27] Genome 10K Community of Scientists Genome 10K: a proposal to obtain whole-genome sequence for 10 000 vertebrate species. J Hered 2009 100 659 674 10.1093/jhered/esp086 19892720 PMC2877544

[B28] WU-BLASTN. http://blast.advbiocomp.com

[B29] Cross-Match. http://www.phrap.org/phredphrap/general.html

