# Supplementary material for: A vertebrate case study of the quality of assemblies derived from next-generation sequences
Source: Genome Biol. 2011 Mar 31;12(3):R31. doi: 10.1186/gb-2011-12-3-r31 (PMC3129681; doi:10.1186/gb-2011-12-3-r31)
Supplement: Additional file 1 — Tables S1 to S3 - sequence coverage and additional assembly results. [file gb-2011-12-3-r31-S1.DOC]

**Supplementary tables**

Table S1. Sequencing reads from different platforms. The 454 reads and Illumina reads are used for *de novo* assemblies. About 2-fold coverage (Q20 bases) of Sanger reads including fosmids and plasmids are added to partial 454 reads (about 8-fold) for the CABOG hybrid assembly.

| Reads type | Raw bases | Q20 Bases | Read number | Average length |
| --- | --- | --- | --- | --- |
| 3730 fosmids | 747,643,121 | 361,552,463 | 760,385 | 983 |
| 3730 plasmids | 3,267,293,591 | 1,856,158,361 | 3,498,792 | 934 |
| FLX 3 kbp paired-ends | 1,227,311,688 | 1,113,317,403 | 8,381,053 | 146 |
| FLX Titanium 20 kbp paired-ends | 1,101,493,971 | 921,041,369 | 5,880,460 | 187 |
| FLX Titanium fragments | 12,547,004,718 | 10,150,753,710 | 37,444,078 | 335 |
| Illumina 200bp paired-ends | 32,858,369,200 | 30,983,530,997 | 328,583,692 | 100 |
| Illumina 300bp paired-ends | 34,739,560,600 | 32,035,934,557 | 347,395,606 | 100 |
| Illumina 2 kbp paired-ends | 13,560,311,600 | 12,423,479,386 | 271,206,232 | 50 |

Table S2. Sequence coverage of Q20 bases for the reference, 454/Newbler and Illumina/SOAP assemblies. The total assembled size about 1.1Gb of the reference assembly is used for coverage estimation.

| Reads type | Coverage (x) |
| --- | --- |
| 3730 plasmids | 5.68 |
| 3730 fosmids | 0.33 |
| 3730 BAC ends | 0.07 |
| FLX 3 kbp paired-ends | 1.01 |
| FLX Titanium 20 kbp paired-ends | 0.84 |
| FLX Titanium fragments | 9.23 |
| Illumina 200bp paired-ends | 28.17 |
| Illumina 300bp paired-ends | 29.12 |
| Illumina 2 kbp paired-ends | 11.29 |

Table S3. Comparative assembly contiguity and accuracy measures of the PCAP 454 *de novo* assembly and CABOG hybrid assembly.

| Metric | 454 (PCAP^a^) | Hybrid (CABOG^b^) |
| --- | --- | --- |
| N50 Contig (kbp) | 9.4 | 32 |
| N50 Supercontig (kbp) | 214 | 3,800 |
| BAC coverage (%) | 97.0 | 96.5 |
| Gene coverage (%) | 93.2 | 91.3 |
| Substitution rate (%) | 0.0195 | 0.0166 |
| Deletion Rate (%) | 0.0532 | 0.0172 |
| Insertion Rate (%) | 0.0112 | 0.0028 |

^a^. PCAP assembly parameters were: pcap.rep.454 -v 14 -w 40 -y 900 -l 120 -s 800; bcontig.rep.454 -y 900 -b 400 -d 60 -f 2 -e 2 -g 4 -h 2 -k 10 -l 35 -i 90 -j 1 -n 22 -o 12000 -p 84 -q 0 -s 1000 -t 3 -v 2 -w 350. Default parameters were used for bdoc.rep.454, bclean.rep.454, and bconsen.454.

^b^. The Q20 sequence coverage for the hybrid assembly is about 8-fold coverage of 454 reads, 1.69-fold coverage of plasmids, and 0.33-fold coverage of fosmids. The cumulative 8-fold coverage of 454 reads is comprised of 1.01-fold coverage of FLX 3 kbp and 0.84-fold coverage of Titanium 20 kbp mate-pair reads, and 5.67-fold coverage of Titanium fragment reads.

CABOG assembly parameters were: useGrid = 1 scriptOnGrid = 0 fakeUIDs = 1 merylMemory = 4000 merOverlapperThreads = 1 merOverlapperSeedBatchSize = 70000 merOverlapperExtendBatchSize = 50000 frgCorrBatchSize = 600000 frgCorrThreads = 1 overlapper = mer unitigger = bog.
