# Supplementary figures and images for: A vertebrate case study of the quality of assemblies derived from next-generation sequences
Source: Genome Biol. 2011 Mar 31;12(3):R31. doi: 10.1186/gb-2011-12-3-r31 (PMC3129681; doi:10.1186/gb-2011-12-3-r31)

## Slide 1
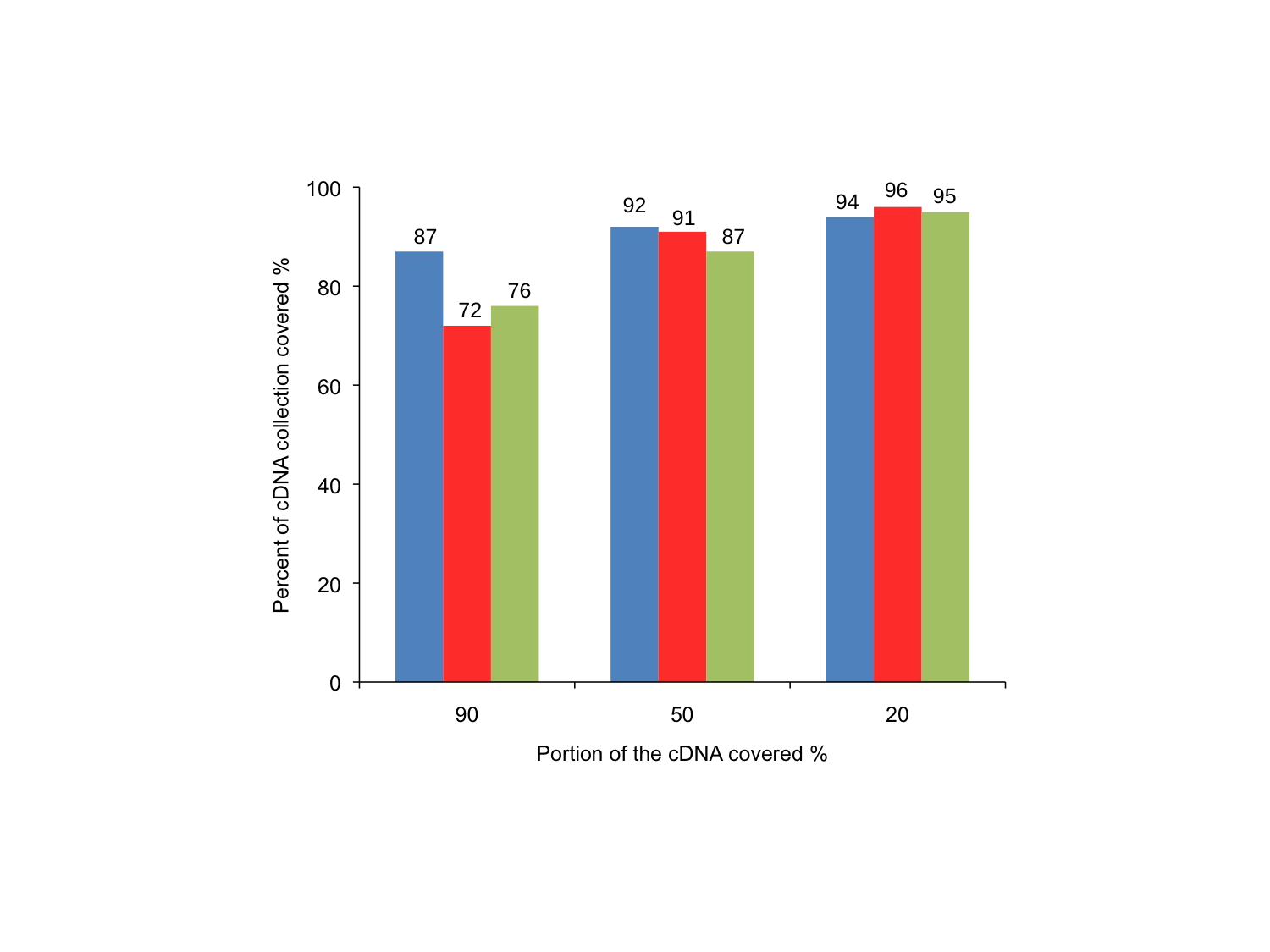

96
95
94
92
91
87
87
76
72

Supplement: Additional file 2 — Figure S1 - coverage of finished cDNAs. Coverage of finished cDNAs (19,626 sequences) as measured using BLAT. A cDNA sequence was considered as sufficiently covered if the percentage identity (95%) and length of the mapped portion was over the indicated cutoff. The alignment length cutoffs shown are 90%, 50%, and 20%. Blue represents the reference, red the 454/Newbler assembly, and green the Illumina/SOAP assembly. [file gb-2011-12-3-r31-S2.PPT]
